# Semen quality pattern and age threshold: a retrospective cross-sectional study of 71,623 infertile men in China, between 2011 and 2017

**DOI:** 10.1186/s12958-019-0551-2

**Published:** 2019-12-09

**Authors:** W. N. Li, M. M. Jia, Y. Q. Peng, R. Ding, L. Q. Fan, G. Liu

**Affiliations:** 1 The Institute of Reproduction and Stem Cell Engineering, Central South University, Luyun Road 8, Changsha, 410008 Hunan China; 2 Reproductive and Genetic Hospital of CITIC-Xiangya, Xiangya Road 84, Kaifu District, Changsha, 410008 Hunan China; 3 Northwest Women’s and Children’s Hospital, Houzaimen Street, Xincheng District, Xi’an, 710000 Shanxi Province China; 4 The Institute of Reproduction and Stem Cell Engineering, Central South University, Luyun Road 8, Changsha, 410008 Hunan China

**Keywords:** Infertile men, Semen parameters, Semen quality, Age threshold

## Abstract

**Background:**

The aim of this study was to provide information on the semen quality pattern of infertile men and age thresholds for semen parameters in China.

**Methods:**

This was a retrospective cross-sectional study investigating 71,623 infertile men from the Reproductive and Genetic Hospital of CITIC Xiangya in Hunan, China, from 2011 to 2017. The Kruskal-Wallis test, Mann-Kendall test, linear regression model and joinpoint regression were used.

**Results:**

Although erratic changes were observed in the median semen parameters (sperm concentration 40.1–52.1 × 10^6^/ml, total sperm count 117.8–153.1 × 10^6^, sperm progressive motility 33.4–38.1%) during the 7 years of observation, no significant decrease in semen quality was found, and 47.88% of infertile men showed normal semen parameters according to the World Health Organization (WHO) criteria. According to the joinpoint regression analysis, sperm progressive motility appeared to decrease earlier than the sperm concentration and total sperm count (at 28, 58, and 42 years of age, respectively).

**Conclusions:**

There is no evidence of a deterioration in semen quality among infertile men in Hunan, China. Semen parameters decreased with increasing age, with turning points noted at different ages. Semen parameters are not absolute evidence for the assessment of male fertility potential. Therefore, we believe that, among semen parameters, the sperm concentration is the best predictor of fertility for ART, followed by motility. Decreased sperm motility may affect natural pregnancy, but it is not necessary for successful IVF.

## Background

Semen quality is a very important factor that reflects male reproductive health. It has been suggested that low semen quality may be a potential contributing factor in reducing fertility rates and increasing number of children born after use of assisted reproductive technology (ART) [1, 2]. However, the question of whether the semen quality of the human population is declining is controversial. Global statistics may be biased and inaccurate partly due to spatial heterogeneity [3] and methodological issues [4]. Bias in the selection of patients may influence the results of published studies [5]. In Carlsen and Swan’s study, publications were excluded if they included men from infertile couples, men referred for oligozoospermia or men selected for either high or low sperm count [6, 7]. The observed decrease in sperm quality was confirmed, but no such decline was found in non-Western countries. Subsequent World Health Organization (WHO) guidelines have included decreasing normal values of these parameters among the population of fertile men since 1980. Literature on the impact of paternal age on semen parameters remains inconclusive [8]. Several studies have suggested that an increase in age is associated with a decline in semen parameters [9–11]. However, other studies [12–15] had conflicting conclusions. As couples delay childbearing, it is becoming increasingly important to determine whether advanced paternal age is associated with diminished semen quality and a higher risk of infertility. China has the largest infertile male population in the world. To our knowledge, no large-scale studies, conducted by a single laboratory, of semen quality of the infertile population in China have been published to date. Therefore, the present study aimed to define a semen quality pattern in infertile men and forge a consensus on age thresholds for semen parameters in China to direct fertility treatment and thereby increase the likelihood of conception.

## Methods

### Study population

This study was a noninterventional retrospective analysis of different subjects. We reviewed the semen analysis database of infertile men, including men who failed to conceive a child over a period of ≥12 months, who visited the Reproductive and Genetic Hospital of CITIC-Xiangya, China, from 1 January 2011 to 31 December 2017. We extracted relevant demographic and clinical information. Demographic information included age, height, weight, body mass index (BMI), smoking and drinking history, the duration of abstinence, and the geographical region of residence. Clinical information included semen parameters and the date of semen analysis. The present study was approved by the ethics committee of the Reproductive and Genetic Hospital of CITIC-Xiangya (No. LL-SC-2018-026).

### Inclusion and exclusion criteria for male partners of infertile couples

Infertility is defined as failure to achieve a clinical pregnancy after 12 months or more of regular unprotected sexual intercourse. Infertility in couples has various causes including male factors, female factors, common factors between partners, and unexplained factors. Due to the changes in the methodology and assessment criteria of semen quality according to WHO guidelines (5th edition) in 2010, only male partners of infertile couples examined from 2011 to 2017 were included. The exclusion criteria were as follows: duplicate records for same sample, incomplete information in the records, men with a history of drug consumption, fever in the previous 6 months, systemic disease, known severe chronic diseases, hypogonadotropic hypogonadism, or treatment with chemotherapeutic agents or radiotherapy, testicular tumours and men living outside Hunan province. The majority of patients (62%) lived in Hunan province.

### Inclusion and exclusion criteria for infertile men

We focused on infertile men who had only male factor infertility (MFI). On the basis of the male partners of infertile couples described above, the inclusion criteria were as follows: men with known causes of infertility or idiopathic infertility (unexplained infertility) or unknown fertility status, such as azoospermia, severe asthenospermia, severe oligozoospermia, severe teratospermia, abnormal acrosome function of sperm; men whose spouses and their ex-husbands had children without fallopian tube blockage; and men who had been treated at other hospitals for conditions specific to males. Because it is difficult to discriminate whether infertility is caused by male factors in many cases and because the current fertility assessment is better for women than for men, we implemented the following exclusion criteria: men with secondary sterility and men who were the male partners of infertile women undergoing ART and who had a known cause of infertility, such as endometriosis, polycystic ovarian syndrome (PCOS), uterine adhesions, uterine deformities (under mediastinal B-mode ultrasound ≥1 cm), obvious hydrosalpinx, fallopian tube blockage, ovulatory dysfunction, chromosomal disorder, or premature ovarian failure (POF).

### Semen analyses

The patients were instructed to abstain from intercourse for 3–5 d. All samples were incubated at 37 °C and analysed within 40 min of collection. The standard WHO (5th edition) guidelines for semen analysis were followed during the 7-year study period. To reduce the intra-assay and interassay variations in the assessment of semen characteristics, all semen analyses were performed by the same four well-trained technicians using the same instrument. Semen volume was estimated by sample weight, assuming that 1 g of semen is equivalent to a volume of 1 ml. The pH value was measured using pH paper and compared with a calibration strip. To determine the concentration of sperm, 5 μl of thoroughly mixed semen was loaded on a Makler counting chamber (Sefi Medical Instruments, Haifa, Israel) under a light microscope at a final magnification of 400×. The number of sperm cells in 10 squares was counted. Sperm motility was graded according to the WHO classification scheme (5th edition) with the following classifications: forward progressive motility (PR), non-progressive motility (NP) and immotility (IM). Semen analysis parameters were extracted from the routine semen analysis reports of the clinical laboratory and included semen volume, sperm concentration, total sperm count, sperm progressive motility, total motile spermatozoa, and immotile spermatozoa. For men who contributed two or more samples, the mean sperm parameters were used for the analysis.

### Morphology

Smears were performed for sperm morphology assessment. Following fixation and Papanicolaou staining using a Baso Papanicolaou staining kit, morphology was assessed according to strict criteria [16]. At least 200 spermatozoa were counted from each sample. The results are expressed as the percentage of normal spermatozoa, head defects, midpiece defects and tail defects.

### Statistical analyses

IBM SPSS Statistics (v. 19.0; IBM Corp., Armonk, NY, USA) was used for the statistical analysis. Baseline characteristics are presented as frequencies (%) for categorical data and as medians and percentiles for non-normally distributed continuous data (semen parameters with markedly skewed distributions). Differences in the median semen parameters across the years of the study were assessed using Kruskal-Wallis analysis of variance, which is a nonparametric test. Trends in semen parameters over the study years were assessed using the Mann-Kendall test. χ2 tests were applied to analyse possible differences in the types of semen between male partners in infertile couples and infertile men. Correlations between semen parameters and age were assessed by Spearman’s rank correlation. We used linear or nonlinear regression models to examine trends in semen parameters. All data were analysed by least-squares linear regression. The semen parameters that correlated with age across the entire age range were then further analysed using the joinpoint regression program 4.5.0.1 (June 2017) from the National Cancer Institute and Information Management Services, Inc. A Monte Carlo permutation test was used to test the significance of turning points (joinpoint). All statistical tests were two-sided, and *p* values < 0.05 were interpreted as statistically significant.

## Results

A total of 198,688 male partners of infertile couples contributed 243,885 data entries between 1 January 2011 and 31 December 2017 (Additional file 1: Table S1). The demographic characteristics of the 71,623 infertile men are summarized in Table 1. No differences were found between the groups in age, height, weight, BMI and abstinence.

**Table 1:** Demographic characteristics of infertile men

Variables	2011(*n* = 6236)	2012(*n* = 8529)	2013(*n* = 10,205)	2014(*n* = 11,809)	2015(*n* = 12,331)	2016(*n* = 11,075)	2017(*n* = 11,438)	*P* value
Age (y)	33(29–37)	33(29–37)	33(29–38)	33(29–38)	33(29–38)	33(29–39)	33(29–39)	0.351
Height(m)	1.69(1.65–1.74)	1.70(1.66–1.76)	1.71(1.66–1.76)	1.69(1.65–1.75)	1.72(1.67–1.76)	1.70(1.65–1.74)	1.71(1.66–1.75)	0.218
Weight(kg)	66.3(59.5–74.0)	70.5(62.5–75.0)	66.2(59.8–74.5)	66.5(60.5–74.8)	69.5(61.5–74.2)	70.1(61.8–75.0)	68.3(61.3–74.3)	0.237
BMI (kg/m^2^)	23.1(21.5–25.0)	24.3(21.9–26.4)	22.5(20.7–24.8)	23.2(21.6–25.2)	23.4(21.8–25.2)	24.2(22.3–25.4)	23.3(21.4–25.0)	0.176
Abstinence(d)	4(3–5)	4(3–5)	4(3–5)	4(3–5)	4(3–5)	4(3–5)	4(3–5)	0.777
Smokers(%)	66.5%	65.2%	58.9%	61.4%	65.6%	62.5%	66.1%	< 0.001
Drinkers(%)	17.3%	18.7%	18.5%	19.6%	20.7%	18.5%	19.3%	< 0.001

*BMI* Body mass index

The semen characteristics of the study populations are presented in Table 2. Among all infertile men, the median values of the semen parameters were higher than or equal to those of the WHO 2010 criteria. Although erratic changes were observed in the median semen parameters during the 7 years of observation (specifically, the sperm concentration varied within 0.5–1.2 million/ml per year), the results clearly suggested the lack of a significant decrease in semen quality (Table 2).

**Table 2:** Characteristics of infertile men’ semen parameters between 2011 and 2017

Year	n	Semen parameters [median (25–75)]						
		Semen volume(ml)	Sperm concentration(10^6^/ml)	Sperm totalcount (10^6^)	Sperm progressive motility (PR)	Total motile spermatozoa[(PR + NP)]	Immotile spermatozoa (IM)	Total motile sperm count (10^6^)
2011	6236	3.2(2.4–4.1)	47.1(20.3–70.9)	143.8(70.1–214.0)	35.5(24.0–46.0)	46.5(38.5–61.9)	53.5(37.8–61.2)	73.8(34.2–117.5)
2012	8529	3.0(2.4–4.2)	51.6(21.0–82.5)	145.3(71.8–178.8)	33.4(25.1–36.9)	49.8(32.3–62.6)	50.2(36.8–68.3)	68.9(30.4–110.1)
2013	10,205	3.2(2.3–4.3)	41.2(18.2–61.3)	126.5(63.0–166.3)	35.2(28.3–37.6)	46.9(41.1–56.8)	53.0(46.1–70.3)	57.6(28.9–108.3)
2014	11,809	3.0(2.2–4.0)	52.1(26.6–85.1)	153.1(72.9–130.7)	35.1(22.1–47.2)	50.3(38.2–61.6)	49.7(38.3–61.8)	75.6(29.9–130.7)
2015	12,331	3.2(2.4–4.2)	40.1(23.5–52.3)	117.8(65.0–178.9)	37.8(28.4–44.9)	46.8(40.2–54.8)	53.2(45.2–59.4)	55.5(26.1–91.9)
2016	11,075	3.2(2.4–4.2)	48.3(28.4–70.2)	145.4(78.5–223.3)	38.1(26.1–46.6)	55.1(42.5–62.5)	44.8(37.3–57.4)	75.4(36.2–123.7)
2017	11,438	3.3(2.5–4.4)	43.6(31.2–59.6)	140.6(82.5–204.9)	36.1(25.5–43.9)	46.7(36.7–56.1)	53.2(43.8–63.1)	64.9(32.5–105.4)
*P*-value^a^		< 0.001	< 0.001	< 0.001	< 0.001	< 0.001	< 0.001	< 0.001
*P*-value^b^		> 0.1	> 0.1	> 0.1	> 0.1	> 0.1	> 0.1	> 0.1
Sen’s slope estimate^c^	Q	0.017	−0.583	−0.533	0.520	0.075	−0.075	− 0.800
	B	3.13	47.10	143.80	34.16	46.75	53.15	69.70

^a^The Kruskal-Wallis test was used to compare the median value between groups

^b^The Mann-Kendall test was used to assess trends in semen parameters from 2011 to 2017

^c^Sen’s slope estimate:equation = Q*(year-firstYear) + B for a linear trend

According to the WHO (4th and 5th edition) standards, the majority of semen volume, sperm concentration and sperm total count measurements were within the accepted normal values (81.17, 67.77, 71.37 and 88.63%, 71.12, 71.58%, respectively), except for sperm progressive motility (31.20 and 70.22%), as presented in Table 3. However, the abnormal rates showed no increase during this time period.

**Table 3:** Percentage of normal semen parameters in infertile men

Parameter	Normal semen parameters according to the recommendations (%) ^a^	2011 (*n* = 6236)	2012 (*n* = 8529)	2013 (*n* = 10,205)	2014 (*n* = 11,809)	2015 (*n* = 12,331)	2016 (*n* = 11,075)	2017 (*n* = 11,438)	2011–2017 (*n* = 71,623)
Semen volume(ml)	WHO (4th)	79.69	81.58	80.66	78.49	82.43	80.79	83.93	81.17
	WHO (5th)	85.17	88.51	85.73	87.58	90.52	89.16	91.75	88.63
Sperm concentration(10^6^/ml)	WHO (4th)	68.94	70.98	65.28	64.98	66.07	67.27	72.16	67.77
	WHO (5th)	70.36	72.13	68.43	68.62	71.51	70.82	75.65	71.12
Sperm total count (10^6^)	WHO (4th)	71.14	71.94	69.52	68.69	71.34	71.02	75.89	71.37
	WHO (5th)	71.29	72.26	69.71	68.84	71.56	71.18	76.11	71.58
Sperm progressive motility (PR) ^b^	WHO (4th)	32.75	33.79	30.85	36.79	28.51	30.47	26.59	31.20
	WHO (5th)	73.22	69.56	68.47	67.56	75.61	69.91	67.89	70.22

^a^Abnormal values of semen parameters were defined by the World Health Organization (WHO) recommendations (4th and 5th). The 4th standards: semen volume < 2 mL, sperm concentration < 20 × 10^6^/mL, sperm total count < 40 × 10^6^, sperm progressive motility(PR) < 50%. The 5th standards: semen volume < 1.5 mL, sperm concentration < 15 × 10^6^/mL, sperm total count < 39 × 10^6^, sperm progressive motility(PR) < 32%

^b^Azoospermia were excluded

**Fig. 1:**
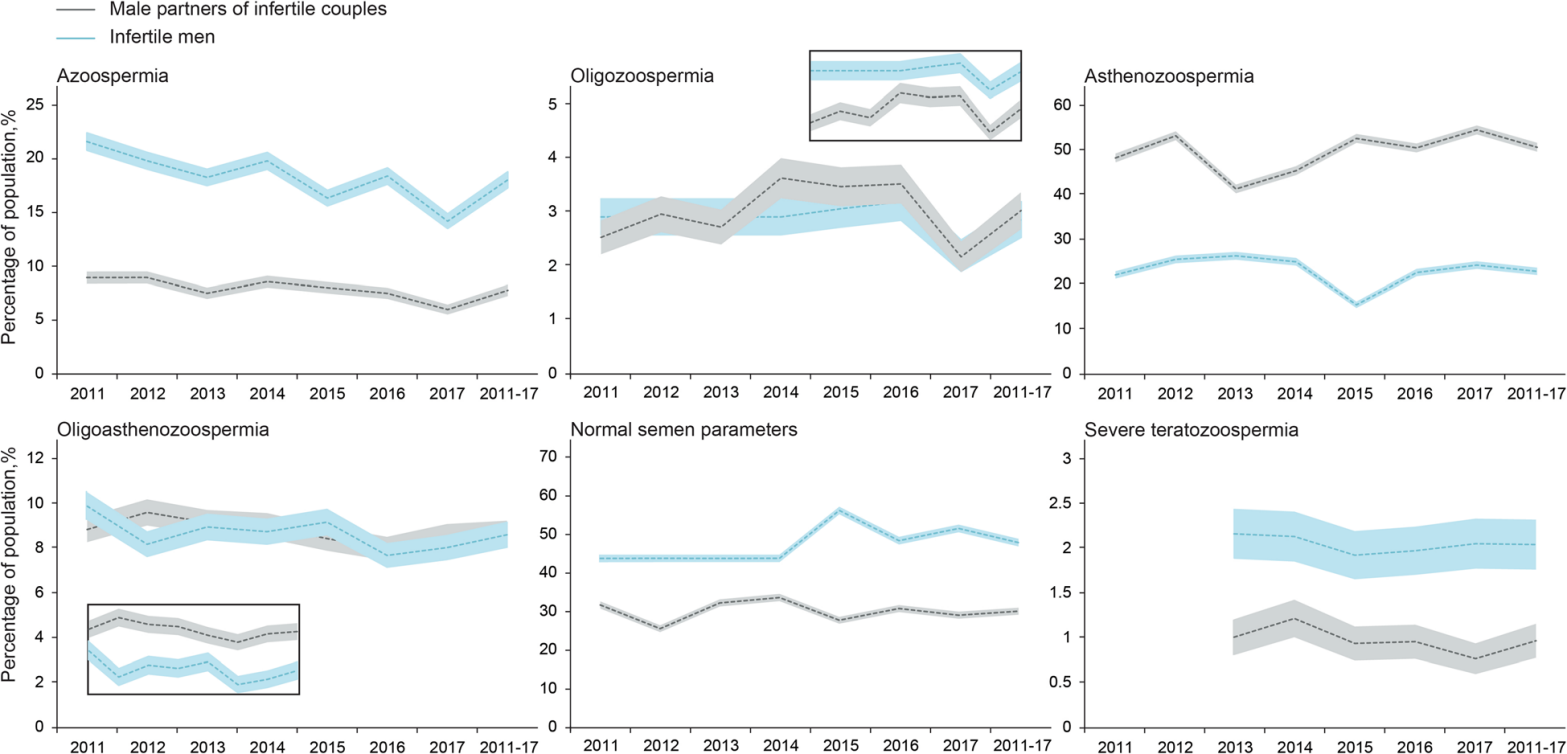
Percentages of different populations among male partners of infertile couples and infertile men between 2011 and 2017. The lines show the percentages, and the shaded areas show the 95% confidence intervals of the percentage curves

Statistically significant differences were found between the male partners of infertile couples and infertile male groups regarding types of semen analysed (Fig. 1, Additional file 1: Table S1; *p* <  0.001) according to the 2010 WHO criteria. The most important differences included the increasing percentages of azoospermia (18.00% vs 7.73%), normal semen parameters (47.88% vs 30.08%), and severe teratozoospermia (2.04% vs 0.96%); a decreased percentage of asthenozoospermia (22.70% vs 50.46%) was found in infertile men compared to male partners of infertile couples.

Spearman’s rank correlation was then performed to explore the possible relationship between semen parameters and age. Table 4 shows the adjusted regression coefficients and *p* values for possible risk factors in relation to semen parameters. The results showed that age was correlated with semen parameters. Linear and nonlinear regression models were used to examine the relationship between age and semen parameters. Five curve estimation results (including linear, logarithmic, reciprocal, second-order polynomial and cubic curve models) across all ages revealed a significant (*p* <  0.001) but weak regression correlation with a poor R squared value.

**Table 4:** Effects of age on semen parameters

Semen parameter	R^2^	coefficient	*P-*value
Semen volume (ml)	0.007	−0.09	< 0.001
Sperm concentration (10^6^/ml)	0.009	0.09	< 0.001
Total sperm count (10^6^)	0	0.02	< 0.001
Sperm progressive motility (PR%)	0.001	−0.03	< 0.001
Total motile spermatozoa [(PR + NP)%]	0.005	−0.07	< 0.001
Immotile spermatozoa (IM%)	0.006	0.08	< 0.001

**Fig. 2:**
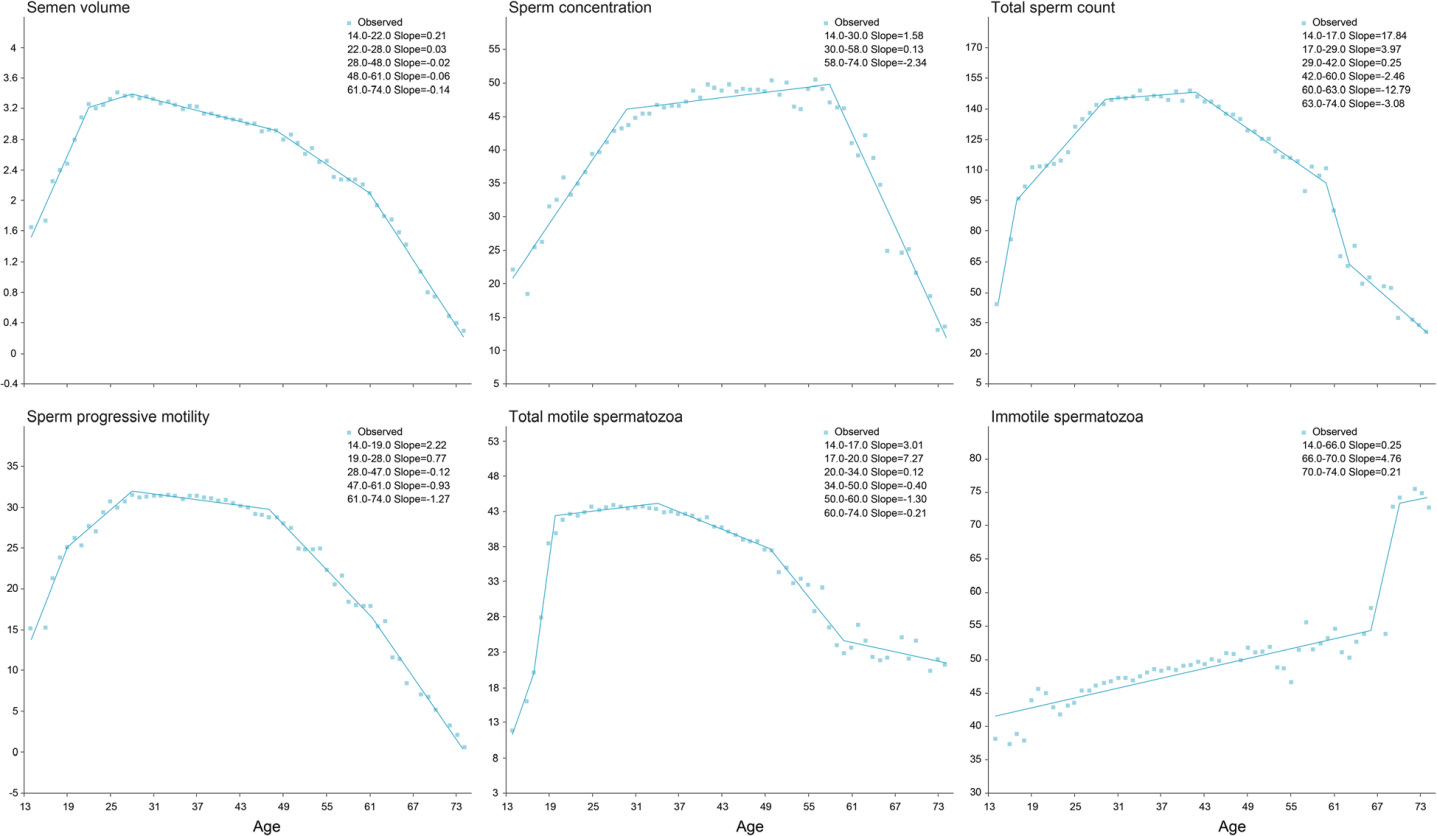
Joinpoint regression analysis of semen parameters with age Values were calculated from the slope of the regression beyond the joinpoint and the average value for each dependent variable at each respective joinpoint

We calculated the joinpoint changes in all semen parameters, which all showed significant declines with age, with the exception of immotile spermatozoa (Fig. 2). The sperm concentration began to decrease rapidly at 58 years of age, declining by 2.34% per year. The total sperm count began to decline slowly at 42 years of age, decreasing by 2.46% per year, and then rapidly declined at age 60 years, by 12.79% per year. Sperm progressive motility slowly decreased beginning at 28 years of age.

## Discussion

To our knowledge, this is the largest study focusing on the semen quality of infertile men, excluding female factors, conducted by a single laboratory in China. This study provides several insights into the semen quality of infertile men in Hunan Province, China, and its variation over time and by age.

The debate on declining semen quality has been ongoing since 1992. The majority of reports indicate a decline in sperm quality in the general population, donors, or infertile men. However, few studies have been conducted on infertile men [17–19]. Carlsen et al. restricted their study to men with proven fertility (39 studies) and “normal” men of unknown fertility (22 studies). When the data were reanalysed, sperm concentration only significantly declined from 1938 to 1972, with no decline in the 20-year period after 1972 [20]. In the present study, surprisingly, no significant decline in the semen quality of infertile men in Hunan, China, was found over the 7-year period studied. The discrepancies between the previous studies and ours may be due to the inclusion of study populations from different time periods, geographic variations, lifestyle-related variables, environmental factors, marital status, socio-economic background, or methodology of semen analysis. Interestingly, the median sperm concentration and total sperm count in the present study (40.1–52.1 × 10^6^/ml and 117.8–153.1 × 10^6^) were not significantly lower than those of young Chinese men (47 × 10^6^/ml, 119 × 10^6^) from the same region examined in 2011–2015 [21] and were different from those of previous studies of infertile men (60.6 × 10^6^/ml [22] and 37.9 × 10^6^/ml [18]). However, the reason for the lack of decline in sperm concentration could not be determined in this study. Possible explanations for effects on sperm production may include lifestyle factors [23], such as the higher prevalence of sedentary behaviour, sleep deficiency, and psychosocial stress among young university students. The data provided no evidence that differences in semen quality differ across geographical regions and showed that lifestyle produced diverse effects on semen quality. It is noteworthy that the median sperm progressive motility value in the present study (33.4–38.1%) is much lower than values obtained in other studies [22] of young Chinese men (50.2 to 43.1%) [21].

Many researchers consider that the reduction in normal reference values in the revised WHO standards (5th edition) [24] indicating the decline in semen quality over the years as a scientific basis. In reality, the WHO-5th reference values for human semen characteristics are significantly different from the WHO-4th reference values in terms of the study population, research methods, observation time, statistical methods and clinical significance. First, in WHO-5th reference, data from 1953 semen samples from five studies in eight countries (not including China) on three continents were combined and analysed; in the WHO-4th reference, Eliasson proposed the reference values based on a single-centre analysis in the 1970s. Second, the studies were prospective (WHO-5th) and retrospective (WHO-4th) studies. Third, in the WHO-5th reference, the semen data were from a study population that had heterogeneous definitions of fertility and had a currently or formerly pregnant partner with time-to-pregnancy (TTP) ≤ 12 months. In the WHO-4th reference, the semen data were from men who had pregnant partners within the first three months. Fourth, median values, which are probably below the arithmetic mean, are more scientific and reasonable because the data distribution of human semen parameters are obviously skewed to the right. The WHO-5th reference uses the 5th percentile as the lower limit of the reference values, while the WHO-4th references uses the arithmetic mean as the point of tangency. Fifth, the WHO-4th reference confirmed that semen parameters were closely related to fertility; the WHO-5th reference reduced the correlation between semen parameters and fertility by substituting reference values for the tangent values and expressing the value as a probability.

The revised WHO standards (5th edition) had the greatest influence on sperm progressive motility, which decreased from < 50 to < 32%. Our data revealed that the proportion of sperm progressive motility within the accepted normal values increased from 31.20 to 70.22%, indicating that large number of infertile men in this study had normal sperm motility (32% < PR < 50%).The WHO provides normal reference ranges for semen parameters. These values are higher than those associated with infertility and reflect the lower range of fertile men. In this study, 47.88% of infertile men had optimal semen quality from a fecundity perspective, which was higher than the rate found in male volunteers in Barcelona (22%) [25] and the general Danish population (25%) [26].

The most influential male infertility guidelines still largely rely upon the concept of abnormal semen analysis to recommend interventions [27]. However, an increasing tendency exists to neglect potentially informative andrological analysis of male partners of infertile couples before opting for ART. The increasing number of male patients undergoing ART does not indicate that male fertility is declining, at least with regard to semen parameters. What is the actual relationship between semen parameters and male fecundity? Approximately 15% of conventional semen analyses do not show obvious anomalies [28]. In our study, the proportion of normal semen parameters in infertile men (47.88%), with possibly unexplained infertility, was higher than that in male partners of infertile couples (30.08%), which supports the fact that semen parameters are indirect, rather than absolute, evidence for the assessment of male fertility potential. Semen parameters can only reflect the probabilities of pregnancy and the minimum sperm quality required for natural pregnancy to a certain extent. They cannot reflect the function of sperm, acrosome activation, acrosome reaction, sperm and egg recognition, or fertilization ability. Noticeably, the proportion of azoospermia was increased and the proportion of asthenozoospermia was decreased in infertile men compared to male partners of infertile couples. Additionally, assessing sperm concentration is a matter of relatively simple counting, while sperm motility is more sensitive to subjective judgement. Therefore, from a clinical perspective, we believe that, among semen parameters, sperm concentration is the best predictor of fertility in ART, followed by motility. Our analysis revealed that decreased sperm motility may affect natural pregnancy, but it was not necessary for successful IVF.

A retrospective analysis reported [11] that sperm concentration declined after 40 years of age, and sperm motility decreased after 43 years of age. Pasqualotto et al. [29] identified age thresholds of > 45 years for sperm concentration and motility. Sperm motility was found to be inversely related to age with peak motility at age < 25 years [30]. Sloter E[31]established an age effect on semen parameters but with no evidence of age thresholds. In this study, we found that advancing age was not related to semen volume but instead was associated with a decrease in total sperm count and sperm progressive motility. In the joinpoint regressions, sperm progressive motility appeared to decrease earlier than sperm concentration and total sperm count (at 28, 58, and 42 years, respectively). That is, sperm motility was statistically more sensitive to aging than other semen parameters. Increasing seminal ROS levels and changes in epididymal and accessory sex gland function may be possible causative factors for the decline in motility with aging [32]. However, the correlation between young age and semen quality was difficult to accurately assess in our study due to the relatively small number of young infertile men (< 21 years). Although the percentages of men aged 41–45 and 46–50 years were increased from 9.26 to 13.64% and from 2.43 to 4.44% in this study, respectively, with the implementation of the second child policy in China, the joinpoint of sperm concentration (at 58 years) was still much higher than that in other reports.

A major strength of this study is the sample size; the inclusion of 71,623 infertile men made this study one of the largest ever conducted in a Chinese population and likely minimized the potential impact of semen sample variability. Second, in this study, men with known causes of infertility or idiopathic infertility (unexplained infertility) or unknown fertility status were included while male partners of infertile women who had a known cause of infertility were excluded. Our results are unique in that they reflect changes in semen quality for infertile men with no/low fertility potential and exclude female factors. Third, the methods, personnel, and instrumentation were consistent throughout the study period, which reduced variation.

However, the key limitations of our study should not be overlooked. First, this was a retrospective analysis that was subject to inherent biases, and the data analysed were relatively crude. Second, this study did not obtain questionnaire data from infertile men. Therefore, we cannot provide strong evidence regarding potential factors known to impact semen quality such as dietary patterns, occupational exposure, lifestyle, education, and socio-economic status. Third, semen analysis was performed using the Makler counting chamber, instead of an improved Neubauer haemocytometer, during the study period. Fourth, we cannot completely distinguish between men with known causes of infertility and those with idiopathic infertility because there is no reason to perform additional nonstandard tests for all patients, such as seminal plasma biochemical detection, chromosomal karyotype analysis, Y chromosome microdeletion (AZF), detection of male infertility-related genes, whole exome sequencing, or epigenetics analysis. Additionally the impact of some diseases (e.g., orchitis, inflammatory obstruction of epididymis, varicocele) on male reproduction varies from person to person, and these diseases do not necessarily lead to male infertility. Fifth, because of the limitations and insufficient understanding in current testing methods for semen, our judgment of female fertility status is better than that of male fertility status. It is difficult to determine whether infertility is caused by male factors in many cases. In our study, we obtained data from infertile men by excluding male partners of infertile women undergoing ART who had a known cause of infertility. Therefore this study did not include the data of male partners of women with unexplained infertility or data from infertile couples with common factors. Sixth, socioeconomic status could not be controlled; the current infertile population was middle-aged and educated, had a higher economic status, lived in Hunan province, and may not be representative of the general population of China.

## Conclusion

In summary, there is no evidence of a deterioration in semen quality among infertile men in Hunan, China. There were increasing percentages of azoospermia and normal semen parameters; a decreased percentage of asthenozoospermia was found in infertile men, compared to male partners of infertile couples, according to the WHO (5th edition) standards.

A total of 47.88% of infertile men showed normal semen parameters. Semen parameters decreased with increasing age, with turning points noted at different ages. Semen parameters are not absolute evidence for the assessment of male fertility potential. We believe that, among semen parameters, the sperm concentration is the best predictor of fertility for ART, followed by motility. Decreased sperm motility may affect natural pregnancy, but it is not necessary for successful IVF. However, these conclusions should be interpreted with caution, and a multi-centre study involving other laboratories is needed to confirm these findings.

## Supplementary information

**Additional file 1: Table S1.** Types of semen in male partners of infertile couples and infertile men.

## Data Availability

The datasets used and/or analysed during the current study are available from the corresponding author on reasonable request.

## Abbreviations

ART: Assisted reproductive technology
BMI: Body mass index
IM: Immotility
NP: Non-progressive motility
PR: Progressive motility
WHO: World Health Organization
