# Supplementary material for: Semen quality pattern and age threshold: a retrospective cross-sectional study of 71,623 infertile men in China, between 2011 and 2017
Source: Reprod Biol Endocrinol. 2019 Dec 9;17:107. doi: 10.1186/s12958-019-0551-2 (PMC6902580; doi:10.1186/s12958-019-0551-2)
Supplement: Supplementary file 1 — Additional file 1: Table S1. Types of semen in male partners of infertile couples and infertile men. [file 12958_2019_551_MOESM1_ESM.docx]

**Table S1** Types of semen in male partners of infertile couples and infertile men

| Variables | Group | 2011 | 2012 | 2013 | 2014 | 2015 | 2016 | 2017 | 2011-2017 |
| --- | --- | --- | --- | --- | --- | --- | --- | --- | --- |
| Azoospermia | 1 | 1453/16328  (8.90) | 2173/24397  (8.91) | 2035/27266  (7.46) | 2586/30321  (8.53) | 2450/30735  (7.97) | 2586/34691  (7.45) | 2078/34950  (5.95) | 15361/198688  (7.73) |
|  | 2 | 1345/6236 (21.57) | 1687/8529 (19.78) | 1860/10205 (18.23) | 2335/11809 (19.77) | 2010/12331  (16.30) | 2033/11075  (18.36) | 1619/11438  (14.15) | 12889/71623  (18.00) |
|  | *P* | < 0.001 | < 0.001 | < 0.001 | < 0.001 | < 0.001 | < 0.001 | < 0.001 | < 0.001 |
| Oligozoospermia | 1 | 412/16328  (2.52) | 719/24397  (2.95) | 740/27266  (2.71) | 1098/30321  (3.62) | 1064/30735  (3.46) | 1219/34691  (3.51) | 754/34950  (2.16) | 6006/198688  (3.02) |
|  | 2 | 181/6236  (2.90) | 247/8529  (2.90) | 296/10205  (2.90) | 342/11809 (2.90) | 376/12331  (3.05) | 352/11075  (3.18) | 249/11438  (2.18) | 2043/71623  (2.85) |
|  | *P* | 0.113 | 0.821 | 0.339 | < 0.001 | 0.033 | 0.094 | 0.914 | 0.022 |
| Asthenozoospermia | 1 | 7847/16328  (48.06) | 12935/24397  (53.02) | 13214/27266  (41.13) | 13715/30321  (45.23) | 16114/30735  (52.43) | 17451/34691  (50.31) | 18990/34950  (54.33) | 100266/198688  (50.46) |
|  | 2 | 1366/6236 (21.91) | 2164/8529 (25.37) | 2670/10205 (26.16) | 2932/11809 (24.83) | 1885/12331 (15.29) | 2485/11075 (22.44) | 2757/11438 (24.10) | 16259/71623  (22.70) |
|  | *P* | < 0.001 | < 0.001 | < 0.001 | < 0.001 | < 0.001 | < 0.001 | < 0.001 | < 0.001 |
| Oligoasthenozoospermia | 1 | 1438/16328  (8.81) | 2234/24397  (9.57) | 2488/27266  (9.12) | 2722/30321  (8.98) | 2582/30735  (8.40) | 2758/34691  (7.95) | 2970/34950  (8.50) | 17192/198688  (8.65) |
|  | 2 | 614/6236  (9.85) | 695/8529  (8.15) | 910/10205  (8.92) | 1028/11809 (8.71) | 1127/12331  (9.14) | 847/11075  (7.65) | 916/11438  (8.00) | 6137/71623  (8.57) |
|  | *P* | 0.016 | 0.005 | 0.544 | 0.391 | 0.014 | 0.311 | 0.103 | 0.495 |
| Normal semen parameters | 1 | 5178/16328  (31.71) | 6236/24397  (25.56) | 8789/27266  (32.23) | 10200/30321  (33.64) | 8525/30735  (27.74) | 10677/34691  (30.78) | 10158/34950  (29.06) | 59763/198688  (30.08) |
|  | 2 | 2730/6236 (43.78) | 3736/8529 (43.80) | 4469/10205 (43.79) | 5172/11809 (43.80) | 6933/12331 (56.22) | 5358/11075 (48.38) | 5897/11438 (51.56) | 34295/71623  (47.88) |
|  | *P* | < 0.001 | < 0.001 | < 0.001 | < 0.001 | < 0.001 | < 0.001 | < 0.001 | < 0.001 |
| Severe teratozoospermia | 1 | NA | NA | 273/27266  (1.00) | 368/30321  (1.21) | 286/30735 (0.93) | 329/34691  (0.95) | 264/34950  (0.76) | 1520/157963  (0.96) |
|  | 2 | NA | NA | 221/10205  (2.16) | 251/11809  (2.13) | 237/12331  (1.92) | 218/11075  (1.97) | 234/11438  (2.05) | 1161/56858  (2.04) |
|  | *P* | NA | NA | < 0.001 | < 0.001 | < 0.001 | < 0.001 | < 0.001 | < 0.001 |

Group 1, male partners of infertile couples; Group 2, infertile men; Severe teratozoospermia, normal morphology≤1%; NA, not available.
